# Distinctive clinical features of early and late-onset Ménière’s disease

**DOI:** 10.3389/fneur.2025.1581670

**Published:** 2025-04-17

**Authors:** Jianwei Lin, Heng Xiao, Chenxin Lin, Gengliang Huang, Xiaojing Guo, Huimin Cai, Shengnan Ye

**Affiliations:** ^1^Department of Otorhinolaryngology-Head and Neck Surgery, The First Affiliated Hospital, Fujian Medical University, Fuzhou, China; ^2^Department of Otorhinolaryngology-Head and Neck Surgery, National Regional Medical Center, Binhai Campus of the First Affiliated Hospital, Fujian Medical University, Fuzhou, China; ^3^Fujian Institute of Otorhinolaryngology, The First Affiliated Hospital, Fujian Medical University, Fuzhou, China

**Keywords:** Ménière’s disease, early-onset, late-onset, DHI, PHQ-9, GAD-7

## Abstract

**Purpose:**

To compare the clinical characteristics of patients with early and late-onset Ménière’s disease (MD) and to investigate the impact of psychological factors between the two groups.

**Methods:**

The patients were divided into two groups based on their age of onset: early-onset (<45 years old) and late-onset (>55 years old). The differences in clinical symptoms, auditory, vestibular examination, gadolinium-enhanced MRI, vertigo, and psychological assessment were compared. To assess the severity of vertigo, the Dizziness Handicap Inventory (DHI) and Visual Analogue Scale (VAS) were used. The Patient Health Questionnaire 9-item (PHQ-9) and the Generalized Anxiety Disorder 7-item (GAD-7) scales were used to assess the patient’s psychological status.

**Results:**

Thirty-five patients were included in the early-onset and thirty-seven in the late-onset MD groups. Tinnitus was more common in the early-onset group. The aggravating (fatigue) and alleviating (ambient quiet; acute rest) factors of a vertigo episode were statistically different between the two groups. The severity of vestibular endolymphatic hydrops, abnormal rate of canal paresis (CP) value of the caloric test, total DHI score, and VAS score were all higher in the late-onset group. PHQ-9 and GAD-7 scores were significantly correlated with total DHI score in the early-onset group.

**Conclusion:**

Early-onset patients have a higher incidence of tinnitus and are more prone to experience vertigo bouts brought on by fatigue. In late-onset patients, vestibular endolymphatic hydrops are more severe, and the vertigo symptoms are more pronounced. Psychological factors are more closely related to the symptoms of vertigo in early-onset patients.

## Introduction

Ménière’s disease (MD) is an inner ear disease characterized by endolymphatic hydrops (1, 2). Recurrent vertigo bouts, fluctuating sensorineural hearing loss, tinnitus, and ear fullness are typical clinical symptoms (3). MD is most common in patients between the ages of 40 and 60, affecting more women than men (4, 5). The incidence occurs in approximately 3.5–513 per 100,000 people and increases with age (6–8). While MD currently remains incurable, the clinical manifestations in patients can be mitigated to some degree through lifestyle modifications, pharmacological interventions, surgical procedures, and vestibular rehabilitation therapy.

The age of onset of MD may differ based on various clinical subtypes. Generally, patients with migraine or autoimmune disorders tend to have an earlier onset compared to those with typical MD and experience more frequent episodes of vertigo (9). In 2021, to analyze the clinical and cytokine characteristics of MD based on the age of onset, Moleon et al. (10) put forward new clinical subtypes of early and late-onset MD. Nevertheless, there are still scarce reports on the comprehensive analysis of the disparities in clinical features between early-onset and late-onset MD patients.

Previous investigations have demonstrated that excessive work, stress, mood fluctuations, sleep disorders, weather variations, and allergic responses can initiate or exacerbate the symptoms of MD (11–13). Clinically, it is observed that the incidence of MD appears to be trending younger. This might be associated with the escalating pressure in the lives and work of young groups, as well as the adverse psychological states such as anxiety, depression, and others caused by long-term overload pressure (14). However, few relevant studies have discussed and analyzed this phenomenon. As a result, this study aimed to compare the clinical characteristics of early-onset and late-onset MD patients and examine the impact of psychological factors between the two groups of patients.

## Materials and methods

### Ethical approval

This study was approved by Fujian Medical University’s Medical Ethics Committee and was performed by the Helsinki Declaration. All subjects provided written informed consent.

### Patients

We retrospectively analyzed the clinical data of all 182 patients who presented to the Otolaryngology-Head and Neck Surgery clinic of our hospital with “vertigo” as the chief complaint from January 2021 to March 2023. MD was clinically diagnosed using diagnostic criteria developed by the Barany Society in 2015 (3). Exclusion criteria were as follows: (i) a history of chronic middle ear disease; (ii) a history of middle or inner ear surgery; (iii) lesions of the central nervous system, the inner ear or cerebellar horn; (iv) a history of traumatic brain injury, stroke, vestibular neuritis/labyrinthitis, or meningitis; (v) age under 18 years old or over 80 years old. There is currently no universal agreement on the definitions of early-onset and late-onset MD. Therefore, based on the grouping methods of other studies, we defined early-onset MD as the first vertigo episode before the age of 45 and late-onset MD as the first vertigo episode after the age of 55 (10, 15). Eventually, Seventy-two patients who were diagnosed with definite unilateral MD and met the above relevant conditions were included in this study.

### Hearing evaluation

Pure tone audiometry was performed on all patients. The audiometer’s model number was OB922 (Madsen, Denmark). All tests were performed by audiologists. In a standard sound insulation shielding room, pure tone hearing thresholds of bilateral ears were measured at 250 Hz, 500 Hz, 1 kHz, 2 kHz, 4 kHz, and 8 kHz using the ascending five and descending ten test method (16). Ultimately, the pure tone average (PTA) across the three frequencies of 0.5 kHz, 1 kHz, and 2 kHz was employed to assess the degree of hearing loss in patients.

### The caloric test

In a dark room with temperatures below 25 degrees Celsius, data was collected using an Ulmer video nystagmography (Synapsys, France) and an ATMOS cold and hot air stimulator (Germany). The patients were supine on the test bed with a 30-degree head-of-bed angle such that the subjects’ horizontal semicircular canal was vertical. Both ears received hot and cold gas stimulation at 25 and 46 degrees Celsius, respectively. Each infusion of gas lasted 40 s. A semicircular canal paresis (CP) value of more than 25% was considered abnormal, indicating that the horizontal semicircular canal’s function was weakened.

### Intratympanic gadolinium injection and MRI acquisition

MRI with gadolinium enhancement was completed within 2 weeks of the patients being diagnosed with MD in our hospital as standard practice. Patients were fully informed of the risks associated with intratympanic injection of contrast agent before signing informed consent and were given the option to choose a unilateral or bilateral injection. All of the patients in this study elected to proceed with bilateral injections. The external auditory canal was sterilized with 75% alcohol, and 1% tetracaine solution was applied to the tympanic membrane. An injection of 0.4 to 0.7 mL of Gd-DTPA diluted 1:8 with normal saline was delivered into the tympanic chamber through a tympanic puncture. A three-dimensional fluid-attenuated inversion recovery (3D-FLAIR) sequence was performed 24 h after the injection of contrast medium on a Siemens Verio 3.0 T MR Scanner (Germany) with a 16-channel head coil.

### Evaluation of endolymphatic hydrops

Endolymphatic hydrops were assessed using the methods previously described by Nakashima (17) and Bernaerts (18). All assessments were performed by two experienced radiologists. In cases where consensus could not be reached, a final decision was made by the more experienced radiologist after discussion. The specific evaluation criteria are shown in Table 1.

**Table 1:** The evaluation criteria of endolymphatic hydrops.

	Cochlea	Vestibule
Grade	No hydrops: No vestibular membrane displacement.	Normal vestibule: The saccule and utricle are visibly separate and take up less than half of the surface of the vestibule.
	Mild hydrops: Vestibular membrane displacement, accompanied by endolymphatic space area no larger than the vestibular scale space.	Hydrops grade I: The saccule has become equal or larger than the size of the utricle but is not confluent with the utricle.
	Significant hydrops: Vestibular membrane displacement accompanied by endolymphatic space area exceeding the vestibular scale space.	Hydrops grade II: There is a confluence of the saccule and utricle with a peripheral rim enhancement of the perilymphatic space.
		Hydrops grade III: The perilymphatic enhancement is no longer visible. There is a complete obliteration of the bony vestibule.

### Assessment scales and questionnaires

The Dizziness Handicap Inventory (DHI) scale was used to assess the degree of dizziness or balance disturbance at the initial presentation of MD. Four metrics can be computed: total score (DHI-T), physical score (DHI-P), emotional score (DHI-E), and functional score (DHI-F). The DHI-T was 100 points, where a higher DHI score was indicative of a more severe degree of dizziness or balance disturbance. Due to the merits of simplicity, rapidity, and maneuverability, the Visual Analogue Scale (VAS) was also employed to assess the severity of vertigo. A scale ranging from 0 to 10 was utilized, where a score of 0 indicated the absence of vertigo, and a score of 10 denoted the most extreme level of vertigo. Participants were asked to select a number between 0 and 10 to rate their peak vertigo severity at the time of the presenting vertigo episode. The assessment of depressive and anxiety symptoms was conducted utilizing the Patient Health Questionnaire 9-item (PHQ-9) and the Generalized Anxiety Disorder 7-item (GAD-7) scales, yielding total scores of 27 and 21 points, respectively. Scores of 5 or higher on the PHQ-9 and GAD-7 typically signify the presence of clinical symptoms associated with depression or anxiety.

### Statistical analysis

All statistical analyses were performed using SPSS 26.0 software. The independent sample *T*-test and Mann–Whitney U test were employed to assess the differences in continuous variables between the two groups, while Fisher’s exact test, Chi-square test, and modified Chi-square test were utilized to evaluate the differences in dichotomous and ordinal variables. The kappa test was employed to evaluate the radiologial inter observer agreement regarding endolymphatic hydrops. The correlation between the partial results was analyzed by the Spearman correlation coefficient. All tests were two-sided, and the statistical significance was set at *p* < 0.05.

## Results

### Characteristics of clinical symptoms

The symptomatic characteristics of the two groups are summarized in Table 2. In the early-onset and late-onset Ménière’s disease groups, there were 35 and 37 patients, respectively. There was no significant difference between the two groups in gender composition, disease duration, duration of each vertigo bout, or number of bouts in the previous 3 months. Tinnitus was more common in the early-onset group than in the late-onset group ( *p* = 0.008). In the analysis of vertigo aggravating factors, nine patients in the early-onset group and three patients in the late-onset group were prone to vertigo exacerbation due to fatigue, with a statistically significant difference ( *p* = 0.045). The early-onset group had a higher symptom remission rate after acute rest ( *p* < 0.001) or eliminating ambient noise ( *p* < 0.001) in the analysis of vertigo-alleviating factors.

**Table 2:** Characteristics of clinical symptoms.

		Early-onset	Late-onset group	*P* value
Number		35	37	
Gender		14M^a^	16M	0.78
Age of first onset (year), mean (SD)		32.2 (8.2)	63.1 (5.2)	<0.001
Disease duration (year), *n* (%)	≤0.5	13 (37.1)	12 (32.4)	0.43
	>0.5,≤1	6 (17.1)	11 (29.7)	
	>1,≤2	3 (8.6)	4 (10.8)	
	>2,≤3	4 (11.4)	6 (16.2)	
	>3	9 (25.7)	4 (10.8)	
Duration of each vertigo attack (hour), *n* (%)	≤1	9 (25.7)	15 (40.5)	0.26
	>1， ≤ 2	8 (22.9)	10 (27.0)	
	>2	18 (51.4)	12 (32.4)	
The number of attacks in the last 3 months, *n* (%)	≤5	22 (62.9)	20 (54.1)	0.73
	>5,≤10	10 (28.6)	13 (35.1)	
	>10	3 (8.6)	4 (10.8)	
Diet, *n* (%)	Normal or light taste	29 (82.9)	32 (86.5)	0.67
	Extreme taste	6 (17.1)	5 (13.5)	
Accompanying symptoms, *n* (%)	Tinnitus	33 (94.3)	26 (70.3)	0.008
	Aural fullness	24 (68.6)	27 (73.0)	0.68
	Nausea	32 (91.4)	30 (81.1)	0.35
	Vomiting	28 (80.0)	29 (78.4)	0.87
	Malaise	19 (54.3)	14 (37.8)	0.16
	Top-heavy	15 (42.9)	11 (29.7)	0.25
	Sweating	15 (42.9)	16 (43.2)	0.97
	Phonophobia	9 (25.7)	12 (32.4)	0.53
	Photophobia	10 (28.6)	15 (40.5)	0.29
Aggravating factors, *n* (%)	Fatigue	9 (25.7)	3 (8.1)	0.045
	Anger	1 (2.9)	1 (2.7)	>0.99
	Noise	1 (2.9)	2 (5.4)	>0.99
	Insomnia	7 (20.0)	5 (13.5)	0.46
Alleviating factors, *n* (%)	Switch off the light	8 (22.9)	4 (10.8)	0.17
	Eliminate ambient noise	21 (60.0)	7 (18.9)	<0.001
	Acute rest	28 (80.0)	13 (35.1)	<0.001
Anamnesis, *n* (%)	Migraine	6 (17.1)	10 (27.0)	0.31
	Carsickness	17 (48.6)	12 (32.4)	0.16
	Hypertension	4 (11.4)	10 (27.0)	0.17
	Diabetes	1 (2.9)	5 (13.5)	0.23

M^a^: Male. *P* ≤ 0.05 was considered statistically significant.

### Audiology and vestibular function examination results

**Figure 1:**
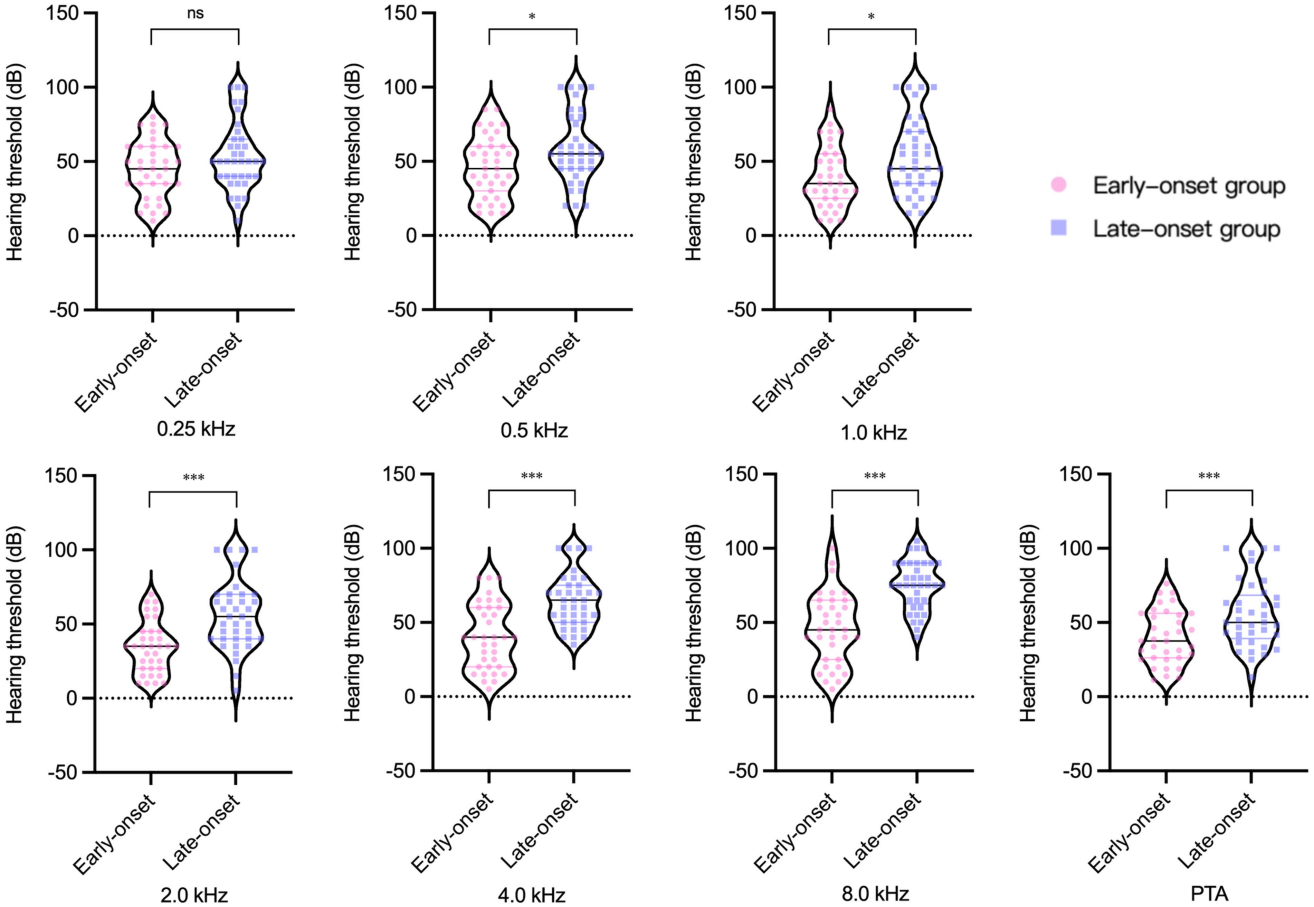
Pure tone audiometry results of the affected ear in two groups of Ménière’s disease. ns, no significance. ^*^ *p* < 0.05,^**^ *p* < 0.01,^***^ *p* < 0.001.

**Figure 2:**
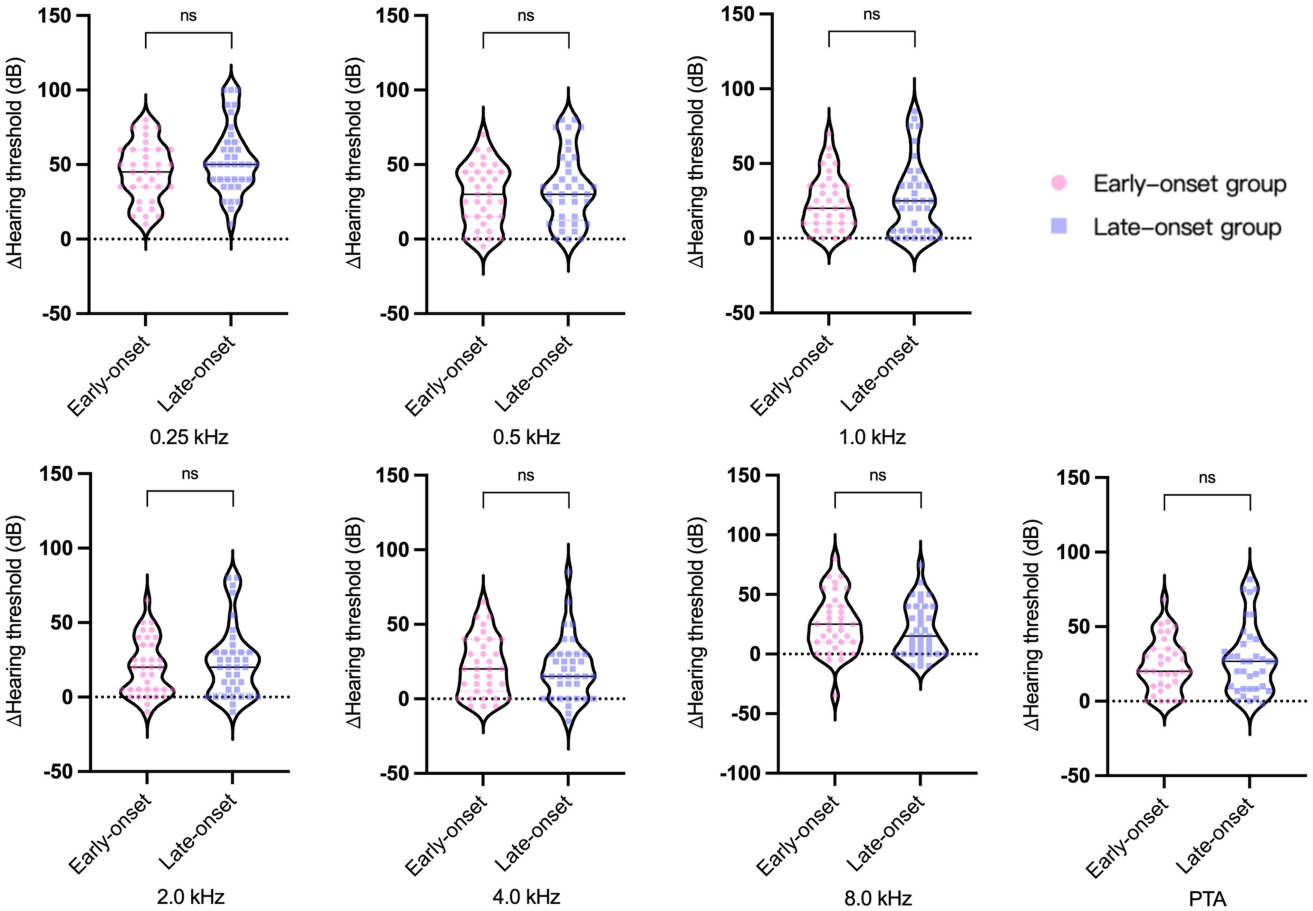
∆PTA and ∆HT across various frequencies between affected and healthy ears within two groups of Ménière’s disease. ns, no significance.

The pure tone audiometry results of the affected ears demonstrated a statistically significant difference between the two groups (Figure 1). The late-onset group had higher PTA and hearing thresholds at 1.0 kHz, 2.0 kHz, 4.0 kHz, and 8.0 kHz than the early-onset group. To mitigate the impact of age-related factors on audiological examination results, we conducted a comparative analysis of the differences in pure tone average (∆PTA) and hearing thresholds (∆HT) across various frequencies between affected and healthy ears within both early and late-onset groups. The findings indicated that there were no statistically significant differences in ∆PTA and ∆HT of each frequency between the two groups (Figure 2). However, the late-onset group had a higher abnormal rate of Cp value (16/37, 43.2%) than that observed in the early-onset group (7/35, 20.0%) ( *p* = 0.035).

### Gadolinium-enhanced MRI results

The grading of cochlear (kappa = 0.875) and vestibular (kappa = 0.942) endolymphatic hydrops demonstrated excellent agreement inter the radiologial observers. In the early-onset and late-onset groups, 6 patients (17.1%) and 4 patients (10.8%), respectively, exhibited neither cochlear nor vestibular endolymphatic hydrops. Among the early-onset and late-onset patients detected with endolymphatic hydrops, there were 10 (28.6%) and 10 (27.0%) patients, respectively, exhibiting mild cochlear endolymphatic hydrops, as well as 13 (37.1%) and 17 (45.9%) patients presenting with significant cochlear endolymphatic hydrops. The degree of cochlear endolymphatic hydrops did not differ significantly between the two groups ( *p* = 0.42). The late-onset group had 5 (13.5%), 13 (35.1%), and 14 (37.8%) cases of grade I, II, and III vestibular endolymphatic hydrops, respectively, while the early-onset group had 11 (31.4%), 15 (42.9%), and 2 (5.7%) cases. The degree of endolymphatic hydrops differed considerably between the two groups ( *p* = 0.005), with the late-onset group showing more vestibular endolymphatic hydrops than the early-onset group. Table 3 displays the detailed data.

**Table 3:** The results of Gadolinium-enhanced MR imaging.

	Grade	Early-onset	Late-onset	*P* value
Cochlea, *n* (%)	No hydrops	12 (34.3)	10 (27.0)	0.72
	Mild hydrops	10 (28.6)	10 (27.0)	
	Significant hydrops	13 (37.1)	17 (45.9)	
Vestibule, *n* (%)	Normal vestibule	7 (20.0)	5 (13.5)	0.009
	Hydrops grade I	11 (31.4)	5 (13.5)	
	Hydrops grade II	15 (42.9)	13 (35.1)	
	Hydrops grade III	2 (5.7)	14 (37.8)	

### Vertigo and psychological assessment scale results

**Figure 3:**
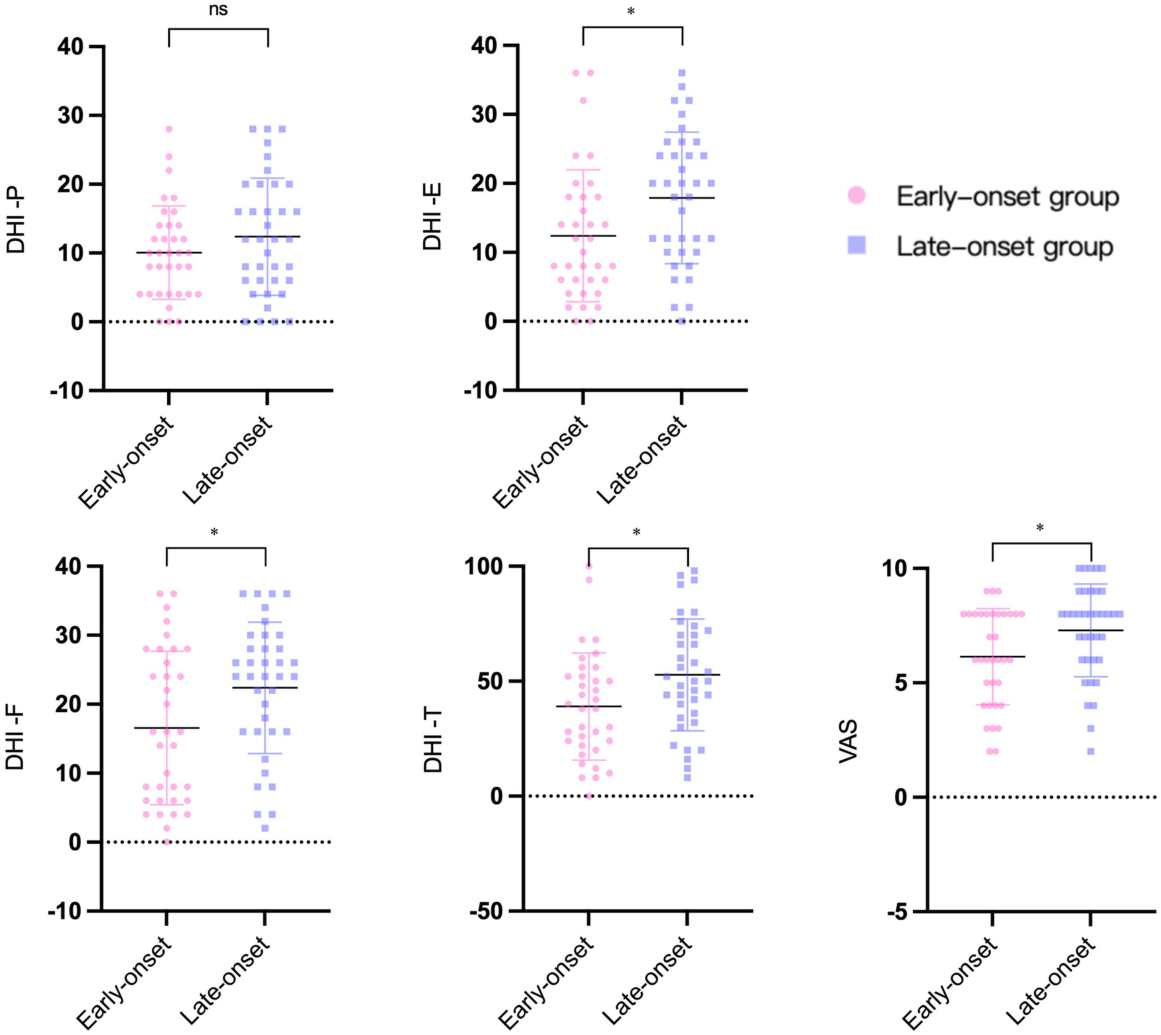
The results of vertigo symptoms assessment scales in two groups. DHI: dizziness handicap inventory. DHI-T, total score. DHI-P, physical score. DHI-E, emotional score. DHI-F, functional score. VAS: visual analogue scale. ^*^ *p* < 0.05. ns: no significance.

**Figure 4:**
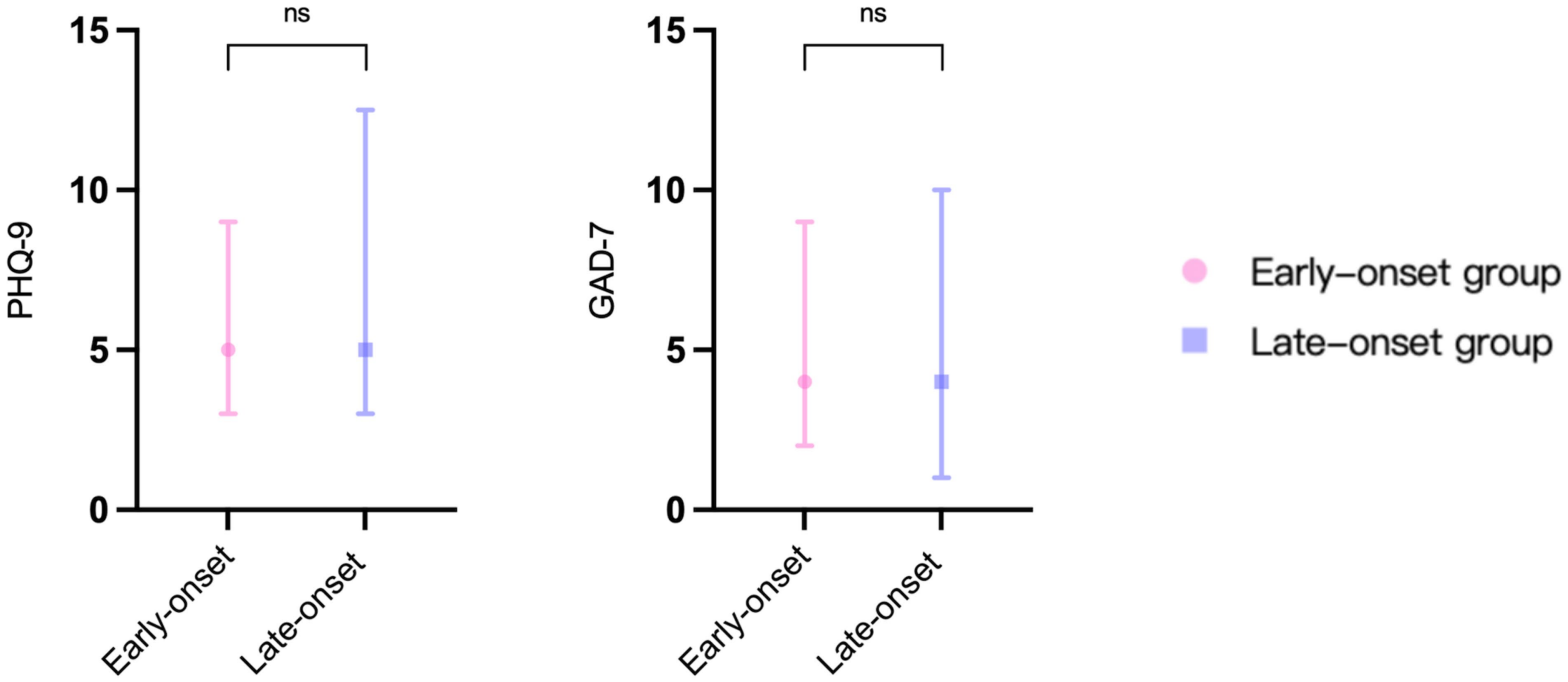
The results of psychological assessment scales in two groups. PHQ-9: patient health questionnaire 9-item scale. GAD-7: generalized anxiety disorder 7-item scale. ns: no significance.

Figure 3 depicts the findings of the two vertigo symptom assessment scales for the two groups. The average scores of DHI-E ( *p* = 0.017), DHI-F ( *p* = 0.02), DHI-T ( *p* = 0.017), and VAS ( *p* = 0.02) in the early-onset group were significantly lower than those in the late-onset group. DHI-P was slightly higher in the late-onset group compared to the early-onset group ( *p* = 0.207), but the difference was not statistically significant. In terms of the psychological assessment scales, the late-onset group’s scores on the PHQ-9 ( *p* = 0.476) and GAD-7 ( *p* = 0.901) were marginally elevated compared to those of the early-onset group, but these differences did not reach statistical significance (Figure 4).

### Correlation between PHQ-9/GAD-7 scale and DHI scores

**Figure 5:**
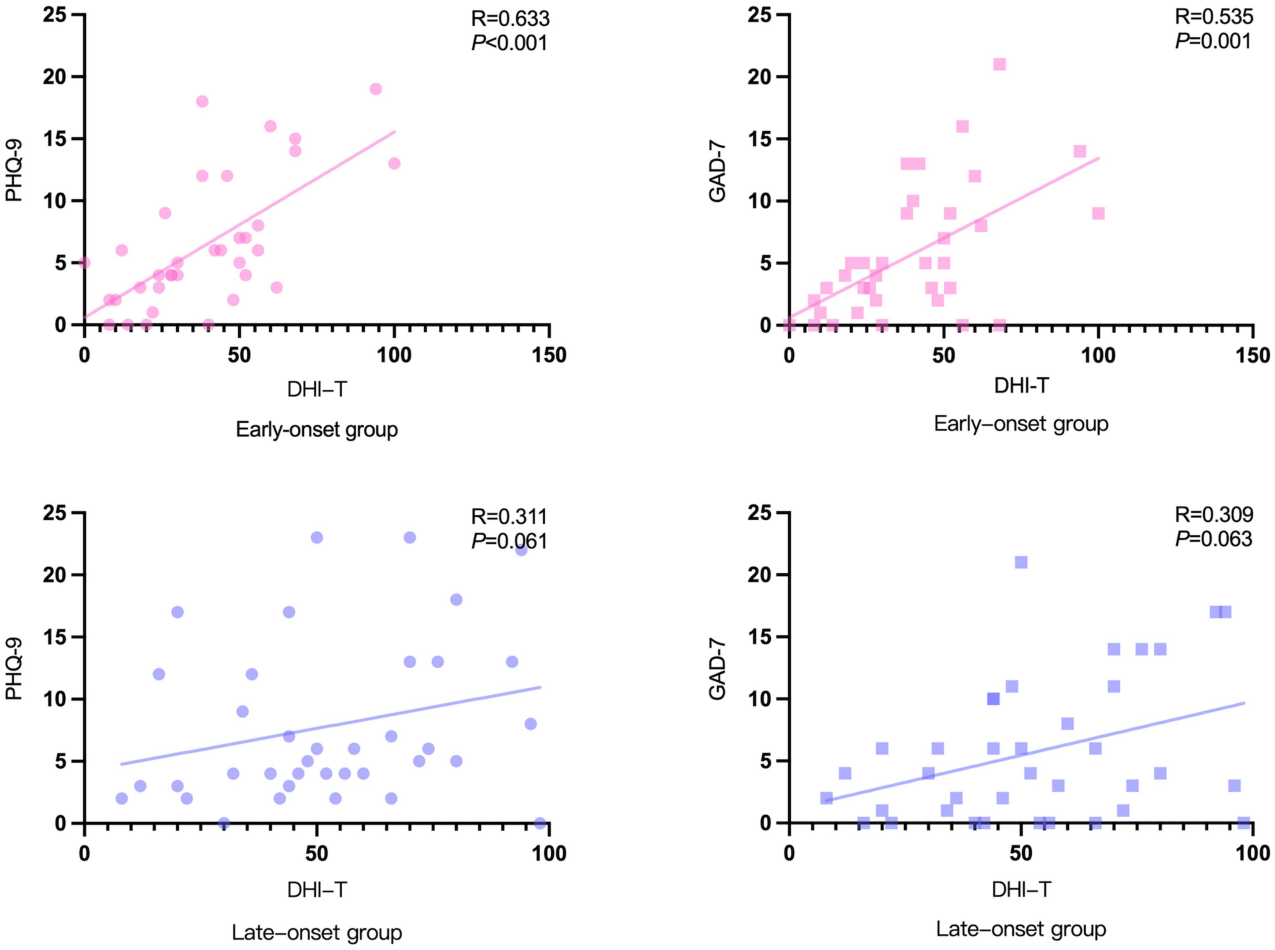
Correlation between PHQ-9/GAD-7 scale and DHI scores.

Since the DHI scale provides a more comprehensive assessment of the degree of vertigo severity, it was selected to analyze the correlation between vertigo symptoms and psychological factors among the two groups of patients (Figure 5). In the early-onset group, there was a correlation between DHI-T and PHQ-9 (R = 0.633, *p* < 0.001) and GAD-7 (R = 0.535, *p* = 0.001) scores. While in the late-onset group, there was no significant correlation between DHI-T and PHQ-9 or GAD-7 scores.

## Discussion

This study primarily examined the clinical symptoms, audiology, vestibular function, and gadolinium-enhanced MRI findings of the inner ear in early-onset versus late-onset Ménière’s disease patients. Additionally, the impact of psychological and emotional factors on the onset of vertigo episodes among both groups was investigated. No statistically significant differences in gender, disease duration, or duration of each vertigo episode were observed between the two groups, 35 early-onset and 37 late-onset MD patients. However, our findings indicate that the two patient groups exhibited distinct differences in symptomatology, imaging characteristics, and psychological status attributable to variations in age phenotypes. This observation underscores the necessity of age-based classification for Ménière’s disease, which may have implications for patient prognosis.

In the clinical symptom analysis, the incidence of tinnitus was higher in the early-onset group than in the late-onset group. Some research has previously linked tinnitus to a disorder of spontaneous nerve activity in the auditory system (19). Tinnitus is thought to be caused by physiological changes in functional inner hair cells. When the cochlea is disrupted by mechanical damage or changes in blood supply, the ion permeability of inner hair cells increases, causing an increase in spontaneous neurotransmitter release from hair cells, further leading to an increase in auditory nerve fiber connection activity (20, 21). We hypothesized that the degree of inner hair cell degeneration in the early-onset group was less severe than in the late-onset group and that the residual secretory function in the early-onset group was stronger, allowing more spontaneous neurotransmitters to be released when a cochlear function was disrupted, resulting in an increased incidence of tinnitus. In addition, it has been proposed that tinnitus is a neurological phenomenon originating in the brain, which arises due to an input deficiency with pacing of higher neural functions (22). The increased prevalence of tinnitus in early-onset patients may also be attributed to the greater neuroplasticity observed in younger individuals.

The findings of this study corroborate that patients in the early-onset group were more likely to have vertigo episodes triggered by fatigue and to have vertigo symptoms relieved by acute rest periods or by silencing ambient noise. Patients in the early-onset group may experience more life and mental stress than those in the late-onset group nowadays, resulting in less sleep or poorer sleep quality than before (23). Furthermore, as a result of the change in lifestyle factors, many young people have developed poor sleep hygiene. These factors collectively make young people more prone to fatigue and can lead to increased sympathetic and parasympathetic dysfunction, potentially leading to MD (24, 25). Patients in the early-onset group in this study experienced greater symptomatic relief after acute rest periods and quieting of ambient noise. As a result, it is necessary to encourage young patients to avoid staying up late and to get enough sleep to reduce the number of vertigo episodes.

Numerous previous studies have established a significant correlation between endolymphatic hydrops and hearing loss (26–28). At the outset, we discovered that the late-onset group had worse overall hearing in the affected ear compared to the early-onset group. To rule out the impact of age on the results, we further compared the difference in ∆PTA and ∆PT at different frequencies between affected and healthy ears within both early and late-onset groups. Our findings revealed no significant difference in the degree of hearing loss caused by MD between the two groups. Combined with the result that there was no significant difference in the degree of endolymphatic hydrops in the cochlea between the two groups, we concluded that the hearing loss resulting from the same degree of endolymphatic hydrops should be comparable between the two groups. The worse hearing outcomes of the affected ear in the late-onset group were largely associated with age-related hearing loss.

In contrast to the results at the cochlear site, the severity of vestibular endolymphatic hydrops was, however, greater in the late-onset group than in the early-onset group, and the difference was statistically significant. We hypothesized that the function of the endolymphatic sac and the inner ear vascular system may gradually decline with age. Late-onset patients exhibit reduced adaptability and are more prone to developing severe vestibular endolymphatic hydrops. At the same time, both the subjective (VAS) and objective (DHI) measures of vertigo symptoms indicated that the late-onset group had significantly more vertigo symptoms than the early-onset group. These two findings show that the presence of vestibular endolymphatic hydrops may impact the severity of vertigo symptoms in MD patients. Consequently, family members should pay more attention to the daily care of patients with late-onset MD to avoid falls, trauma, and other accidents resulting from vertigo. The caloric test is an objective examination used to determine the function of the horizontal semicircular canal. The results of Choi and Zhang’s research showed a strong relationship between the degree of vestibular endolymphatic hydrops and the CP value from the caloric test (29, 30). In the present study, the rate of abnormal CP values was significantly higher in the late-onset group than in the early-onset group. We hypothesized that endolymphatic hydrops might lead to decreased compliance of the membranous labyrinth and disorders of the endolymphatic ion concentration, which could result in decreased responsiveness of the horizontal semicircular canal to external stimuli (e.g., cold and heat changes), increasing the rate of abnormal CP values.

Although the early-onset group’s vertigo symptoms were less severe than the late-onset group’s, we found a significant correlation between vertigo symptoms and psychological scores in the early-onset group, which supported our original hypothesis that psychological problems caused by a fast-paced lifestyle and greater work pressures may disproportionately impact younger MD patients. Furthermore, severe vertigo symptoms can cause low mood or aggravate psychological depression. Long-term mental stress and depression can amplify the subjective feeling of vertigo and may also increase the frequency of MD through sympathetic and parasympathetic disorders, both of which create a vicious circle between vertigo and psychological depression (24).

The limitation of this study included the relatively small sample size. We hope that future studies will increase the sample size and follow up with MD patients to report any differences in the management and recurrence of vertigo symptoms, hearing, and psychological status between the two groups after formal treatment.

## Conclusion

Early-onset MD patients have a higher incidence of tinnitus than late-onset patients, and they are more likely to have vertigo bouts caused by fatigue. In these patients, remission is more easily achieved after acute rest periods or silencing of ambient noise. In late-onset patients, vestibular endolymphatic hydrops are more severe, the abnormal rate of CP value is higher, and the vertigo symptoms are more pronounced. Psychological factors are more closely related to the symptoms of vertigo in early-onset patients, suggesting that timely psychological intervention may be beneficial for the management of vertigo.

## Data Availability

The raw data supporting the conclusions of this article will be made available by the authors, without undue reservation.
